# Novel Biobased Double Crystalline Poly(butylene succinate)-*b*-poly(butylene 2,5-thiophenedicarboxylate) Multiblock Copolymers with Excellent Thermal and Mechanical Properties and Enhanced Crystallization Behavior

**DOI:** 10.3390/polym17040450

**Published:** 2025-02-08

**Authors:** Haidong Yang, Shiwei Feng, Zhaobin Qiu

**Affiliations:** State Key Laboratory of Chemical Resource Engineering, Beijing University of Chemical Technology, Beijing 100029, China; 2023200346@grad.buct.edu.cn (H.Y.); 2023400126@grad.buct.edu.cn (S.F.)

**Keywords:** biobased polyester, poly(butylene succinate), poly(butylene 2,5-thiophenedicarboxylate)

## Abstract

Novel biobased double crystalline poly(butylene succinate)-*b*-poly(butylene 2,5-thiophenedicarboxylate) (PBS-*b*-PBTh) multiblock copolyesters with excellent thermal and mechanical properties were prepared from two hydroxyl-terminated PBS-diol and PBTh-diol prepolymers via a chain extension reaction. Both PBS and PBTh segments were semicrystalline, with the aliphatic PBS segment being the soft segment while the aromatic PBTh segment was the hard segment. In the case of PBS-*b*-PBTh, the two segments were partially miscible in the amorphous region; moreover, the melting temperature of each segment still remained very high compared with that of each homopolyester PBS and PBTh. The melt crystallization behavior of both segments was enhanced in the case of PBS-*b*-PBTh, which was attributed to different mechanisms. The crystal structure study revealed that both segments crystallized separately and showed the characteristic diffraction peaks, respectively. Compared with that of PBS, PBS-*b*-PBTh displayed a significant increase in the elongation at break while still maintaining a relatively high break strength. This research provides some new insights to synthesize biobased polyesters with excellent thermal and mechanical properties, which should be interesting from a sustainable viewpoint.

## 1. Introduction

The over exploitation of petrochemical resources has caused serious environmental issues; therefore, biodegradable polymers obtained from renewable resources have attracted great attention. Poly(butylene succinate) (PBS) has been regarded as one of the most promising fully biodegradable aliphatic polyesters. PBS is produced from succinic acid (SA) and 1,4-butanediol (BDO), which has a high melting point (*T*_m_) of 114 °C, good thermal stability, and excellent mechanical property [1,2,3,4]. The mechanical property of PBS is close to that of low-density polyethylene; therefore, it may find many end uses in various fields. However, the disadvantages of PBS such as the slow rate of biodegradation, poor toughness, and poor gas barrier have limited its practical application [5,6,7,8,9,10,11,12].

Copolymerization is an efficient method to modify the chemical structure and physical properties of PBS. For instance, the following units have been introduced to the main chain of PBS through a copolymerization process, such as ethylene succinate, butylene azelate, *ε*-caprolactone, propylene succinate, and hexamethylene succinate, to improve the toughness and adjust the biodegradation rate [13,14,15,16,17]. In addition, the thermal and mechanical properties of PBS can be further improved by copolymerizing with aromatic monomers. For instance, poly(butylene succinate-*co*-butylene terephthalate) (PBST), which has recently been commercialized in China, shows excellent gas barrier and mechanical properties [18,19]. Lv et al. reported that the addition of dimethyl 2,5-furandicarboxylate into PBS for copolymerization significantly improved the elongation at break because the addition of the furan ring reduced the crystallinity of PBS, thereby facilitating its enzymatic degradation [20].

2,5-Thiophenedicarboxylic acid (TDCA) is an emerging biobased aromatic diacid that can be utilized in the synthesis of polyesters with specific functionalities. The structure of TDCA primarily differs from that of 2,5-furandicarboxylic acid (FDCA) in the presence of a sulfur (S) atom or an oxygen (O) atom on the five-membered aromatic ring [21]. Because of the low electronegativity of the S atom, TDCA is closer in aromaticity and rigidity to terephthalic acid (TPA) than FDCA [22]. So far, some TDCA-based polyesters have been reported in the literature such as poly(ethylene 2,5-thiophenedicarboxylate) (PETh), poly(butylene 2,5-thiophenedicarboxylate) (PBTh), poly(propylene 2,5-thiophenedicarboxylate) (PPTh), and poly(pentamethylene 2,5-thiophene dicarboxylate) (PPeTh) [23,24,25,26,27,28,29]. Some TDCA-based copolyesters exhibited excellent thermal, mechanical, and gas barrier properties; moreover, they even showed good biodegradability depending on the detailed chemical structure and composition of aliphatic comonomers [30,31,32,33,34,35]. For instance, Zhu et al. reported that poly(butylene succinate-*co*-butylene 2,5-thiophenedicarboxylate) (PBSTh) copolyesters displayed better mechanical properties, gas barrier, and biodegradability [34]. The elongation at break of the copolyesters was increased to 523%. The CO_2_ and O_2_ barrier as well as degradation rate of PBSTh were superior to those of poly(butylene adipate-*co*-terephthalate) (PBAT). Wang et al. synthesized PBSTh copolyesters with different molar percentages of TDCA. PBSTh29 (BTh unit = 29 mol%) showed a very high elongation at break to 1060 ± 60%; however, the *T*_m_ of PBSTh29 significantly decreased to 86.3 °C from 113.6 °C for PBS [35].

It is obvious that the elongation at break of PBS-based random copolymers may be significantly improved; however, the *T*_m_ values also remarkably decrease. In order to increase the elongation at break and toughness while still maintaining a relatively high *T*_m_, the synthesis of PBS-based multiblock copolymers is an efficient method [36,37,38,39,40]. Multiblock copolymers show a relatively high molecular weight and excellent mechanical properties than random copolymers [36,37,38,39,40]. In previous studies, we once synthesized poly(butylene succinate)-*b*-poly(butylene sebacate) (PBS-*b*-PBSe) multiblock copolyesters and poly(butylene succinate)-*b*-poly(diethylene glycol terephthalate) (PBS-*b*-PDEGT) multiblock copolyesters with relatively high *T*_m_ values and improved the mechanical properties [38,39]. In the case of PBS-*b*-PBSe, both the PBS and PBSe segments were crystalline with the PBS segment being the hard segment and the PBSe segment being the soft segment; moreover, the *T*_m_ of the PBS block was higher than that of the PBSe block [38]. In the case of PBS-*b*-PDEGT, the PBS segment was crystalline while the PDEGT segment was amorphous; moreover, the PBS segment was the soft segment while the PDEGT segment was the hard segment [39]. It should also be noted that both the PBS and PBSe segments were biobased and biodegradable while the PDEGT segment was fossil based and nonbiodegradable [38,39].

In this research, novel PBS-*b*-PBTh multiblock copolyesters were successfully synthesized from PBS-diol and PBTh-diol prepolymers using hexamethylene diisocyanate (HDI) as the chain extender to improve the mechanical property (mainly the elongation at break) while still maintaining the relatively high *T*_m_ of the PBS segment. In the case of PBS-*b*-PBTh, both the PBS and PBTh segments were crystalline, with the PBS segment being the soft segment and the PBTh segment being the hard segment; moreover, the *T*_m_ of the PBTh segment was higher than that of PBS. In addition, both the PBS and PBTh segments were biobased, although the PBS segment was biodegradable while the PBTh segment was nonbiodegradable. The incorporation of the PBTh segment not only improved the elongation at break of the PBS segment, but also accelerated the crystallization of the PBS segment. The results should be interesting and important in the field of biobased polymers from a sustainable viewpoint.

## 2. Experimental

### 2.1. Materials

TDCA (98%) was purchased from Zhengzhou Alfa Chemical Co., Ltd. SA (99.5%) (Zhengzhou, China) and BDO (99.5%) was bought from Tianjin Fuchen Chemical Reagent Co., Ltd. (Tianjin, China). The catalyst tetrabutyl titanate (TBT, 99%) was obtained from Beijing Changping Jingxiang Chemical Factory (Beijing, China). The chain extender HDI was supplied from Shanghai McLean Biochemical Technology Co., Ltd. (Shanghai, China).

### 2.2. Synthesis of PBS-Diol and PBTh-Diol Prepolymers

**Figure 1:**
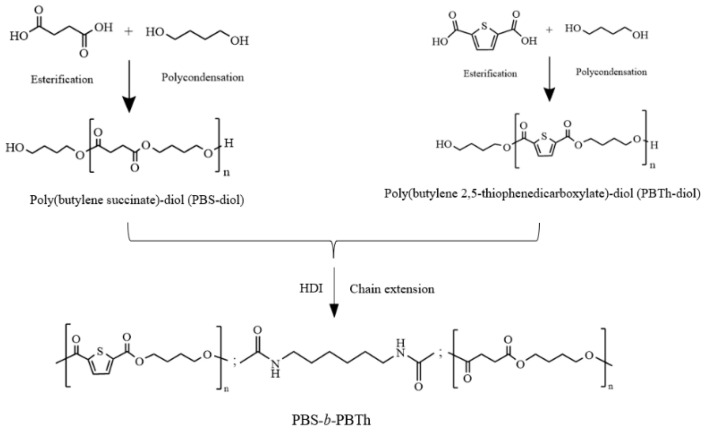
Synthesis routes of PBS-diol, PBTh-diol, and PBS-*b*-PBTh.

Figure 1 illustrates the synthetic procedure for the two prepolymers. PBS-diol was synthesized through a conventional two stage process involving esterification and polycondensation, similar to the method described by Zeng et al. [41] and used in our previous studies [38,39]. PBTh-diol was also synthesized using a similar procedure, which was described in detail in our previous research [26].

### 2.3. Synthesis of PBS-b-PBTh Multiblock Copolymers

As shown in Figure 1, PBS-*b*-PBTh multiblock copolymers were synthesized from PBS-diol and PBTh-diol prepolymers using HDI as the chain extender. To achieve high molecular weight multiblock copolyesters, the molar ratio of HDI to the total moles of the two prepolymers was 1:1. The mass ratios of PBS-diol to PBTh-diol were set as 100:0, 80:20, 60:40, and 0:100, and the corresponding samples were named as PBS, PBS-*b*-PBTh2, PBS-*b*-PBTh4, and PBTh, respectively. A predetermined amount of PBS-diol and PBTh-diol was added into a three-neck flask. Then, the mixture was heated to 160 °C under a nitrogen atmosphere. HDI was then added to the flask. Vigorous stirring and reaction were carried out for 1 h to synthesize the multiblock copolyesters.

### 2.4. Characterization

The chemical structure and composition were determined on a Bruker AV600 hydrogen nuclear magnetic resonance (^1^H NMR) for PBS-diol, PBTh-diol, and PBS-*b*-PBTh using deuterated chloroform (CDCl_3_) as the solvent.

The intrinsic viscosity [*η*] values of all of the synthesized polymers were measured in a mixture of phenol and 1,1,2,2-tetrachloroethane (V/V = 1:1) at a concentration of 0.2 g/50 mL on a Ubbelohde viscometer with a capillary radius of 0.3–0.4 mm at 25 °C.

The thermal stability of all samples was determined with thermogravimetric analysis (TGA) (TA Instrument Q50, New Castle, DE, USA). For the TGA test, about 10 mg of sample was placed in an aluminum pan, which was further placed in a platinum crucible. The experiments were carried out under a nitrogen atmosphere from room temperature to 580 °C at a rate of 20 °C/min.

The basic thermal parameters and non-isothermal crystallization behavior were investigated on a differential scanning calorimeter (DSC) (TA Instrument DSC Q100) with a nitrogen flow of 50 mL/min. To determine the glass transition temperature (*T*_g_), the samples (about 5 mg) were heated to 180 °C at 40 °C/min and maintained for 3 min to eliminate the thermal history. The sample was then quenched at a fast cooling rate of 60 °C/min to −60 °C and then heated to 180 °C at 10 °C/min. For the melt crystallization behavior study, a heating–cooling–reheating process was applied with a rate of 10 °C/min. The melt crystallization temperature (*T*_cc_) was read from the cooling process, while the *T*_m_ was read from the second heating process.

The crystalline morphology of the samples was studied by means of a polarizing optical microscope (POM) (Olympus BX51) with a temperature controller (Linkam THMS 600, Redhil, UK).

Wide angle X-ray diffraction (WAXD) experiments were carried out on a Rigaku Ultima IV X-ray diffractometer (CuKα radiation, λ = 1.5406 nm, 40 kV, and 200 mA) from 5° to 50° at a rate of 5 °/min. All samples were first pressed into thin films of about 1 mm in thickness at 160 °C and then crystallized in an oven at 85 °C for 12 h.

The tensile mechanical properties were measured on a universal tensile testing machine (UTM5205XHD from SUNS, Zhangzhou, China) with a cross head rate of 20 mm/min at room temperature. The samples were prepared and cut by a hot pressing process. The size of the dumbbell shaped specimens was 0.5 mm in thickness, 4.0 mm in width, 50.0 mm in length, and 20.0 mm in a standard distance. The average data were obtained from at least three tests.

## 3. Results and Discussion

### 3.1. ^1^H NMR and Intrinsic Viscosity Studies

**Figure 2:**
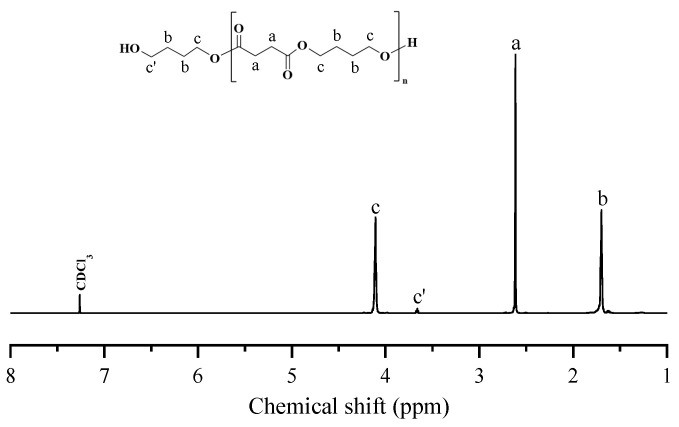
Chemical structure and ^1^H NMR spectrum of PBS-diol.

**Figure 3:**
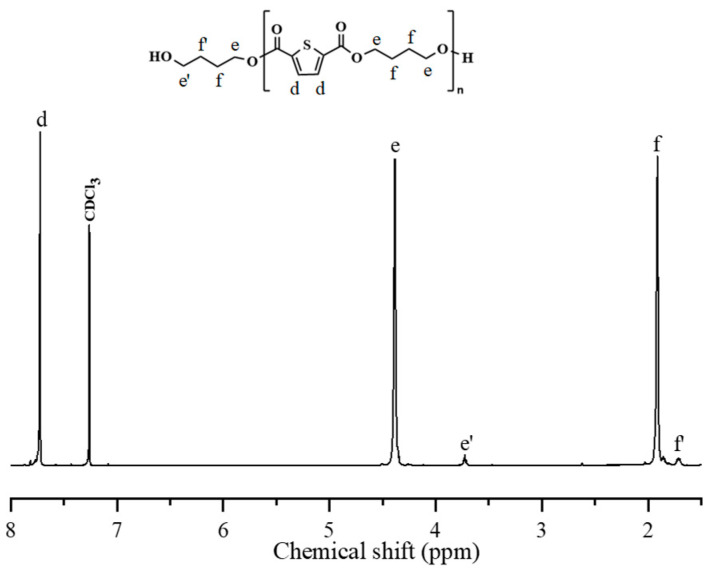
Chemical structure and ^1^H NMR spectrum of PBTh-diol.

**Figure 4:**
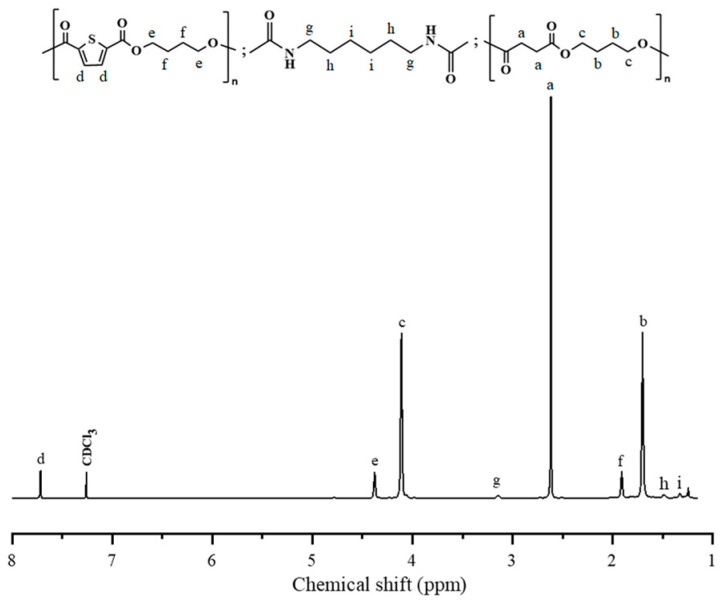
Chemical structure and ^1^H NMR spectrum of PBS-*b*-PBTh2.

The chemical structures of PBS-diol, PBTh-diol, and PBS-*b*-PBTh were first analyzed and confirmed by ^1^H NMR. The ^1^H NMR spectra of PBS-diol and PBTh-diol are shown in Figure 2 and Figure 3, respectively, while that of PBS-*b*-PBTh2 is illustrated in Figure 4. As seen in Figure 2, the signal at 2.62 ppm (δH*^a^*) was attributed to the methylene from SA, while the proton peaks located at 1.70 ppm (δH*^b^*) and 4.11 ppm (δH*^c^*) corresponded to the methylene from BDO. The proton signal at 3.66 ppm (δH*^c^*^’^) corresponded to the first methylene group next to the hydroxyl group at the end of the chain segment. The number-average molecular weight (*M*_n_) of PBS-diol was calculated by Equation (1), which was determined to be 4150 g/mol.

(1) $$M_{n,\mathrm{PBS}\text{-}\mathrm{diol}}=90+172\times \frac{I_{4.11}}{I_{3.66}}$$

where *I*_4.11_ and *I*_3.66_ represent the proton signals at the 4.11 ppm (δH*^c^*) and 3.66 ppm (δH*^c^*^’^) integral areas, respectively; 172 and 90 g/mol are the molecular weights of the repeating unit of PBS and end group of BDO, respectively.

Figure 3 displays the chemical structure of the PBTh-diol prepolymer. Different from that of PBS-diol, a strong and obvious signal located at 7.72 ppm (δH*^d^*) appeared in PBTh-diol, corresponding to the proton on the thiophene from TDCA. The proton signals located at 4.38 ppm (δH*^e^*) and 1.92 ppm (δH*^f^*) were from the methylene group of BDO. The signal at 3.72 ppm (δH*^e^*^’^) arose from the first methylene group next to the hydroxyl group at the end of the chain segment, while the signal at 1.72 ppm (δH*^f^*^’^) represented the second methylene group near the terminal hydroxyl group. The *M*_n_ of PBTh-diol was calculated by Equation (2).

(2) $$M_{n,\mathrm{PBTh}}\text{-}\mathrm{diol}=\frac{I_{4.39}}{I_{3.74}}\times 226+90$$

where *I*_4.39_ and *I*_3.74_ correspond to the integral areas of the proton signal at the 4.39 ppm (δH*^c^*) and 3.74 ppm (δH*^c^*^’^) integral areas; 226 and 90 g/mol are the molecular weights of the repeating unit of PBTh and BDO, respectively. As a result, PBTh-diol displayed an *M*_n_ of 6503 g/mol after the calculation.

As described in the experimental section, two PBS-*b*-PBTh block copolymers with different segment ratios were synthesized from PBS-diol and PBTh-diol using HDI as the chain extender; moreover, the two homopolymers PBS and PBTh were also synthesized from PBS-diol and PBTh-diol using a similar method, respectively. Figure 4 shows the ^1^H NMR spectrum and the chemical structure of PBS-*b*-PBTh2 as an example. As seen in Figure 4, the proton signals located at 3.14 ppm (δH*^g^*), 1.49 ppm (δH*^h^*), and 1.33 ppm (δH*^i^*) were from the methylene group of HDI. The disappearance of hydroxyl proton signals at the end of the PBTh-diol and PBS-diol chain segments was confirmed, indicating the complete reaction of terminal hydroxyl groups during the chain-extension reaction process. Furthermore, the contents of each chain segment in the multiblock copolyester were calculated through Equations (3)–(5) by analyzing the corresponding position of each proton signal and its integral area:

(3) $$F_{\mathrm{PBTh}}=\frac{226\times 2I_{7.72}}{170\times I_{3.14}+172\times I_{2.62}+226\times {2I}_{7.72}}$$

(4) $$F_{\mathrm{PB}S}=\frac{172\times I_{2.62}}{170\times I_{3.14}+172\times I_{2.62}+226\times {2I}_{7.72}}$$

(5) $$F_{\mathrm{HDI}}=1{-F}_{\mathrm{PBTh}}-F_{\mathrm{PBS}}$$

where *I*_7.72_, *I*_2.62_, and *I*_3.14_ are the integrated area of the proton from TDCA, SA, and HDI, respectively; 170, 226, and 172 g/mol are the molecular weights of HDI and the repeating units of PBTh and PBS, respectively. The calculated data are summarized in Table 1. From Table 1, *f* and *F* were basically the same, indicating that the composition of PBS-*b*-PBTh block copolyesters could be controlled by adjusting the feeding ratio to achieve the desired properties. In brief, PBS*-b*-PBTh multiblock copolyesters were successfully synthesized.

The intrinsic viscosity [*η*] was obtained by the commonly used one-point method [42]. The [*η*] values of PBS, PBS-*b*-PBTh, and PBTh were calculated by Equation (6) and are also presented in Table 1.

(6) $$[\eta ]=\frac{\sqrt{2(\eta_{\mathrm{sp}}-\ln\eta_{r})}}{c}$$

where *η*_sp_ represents the specific viscosity, *η*_r_ represents the relative viscosity, and *c* is the concentration of the solution to be measured.

The [*η*] values of all samples were above 0.9 dL/g, indicating that they may show relatively high molecular weights and good mechanical properties. It should be noted that the mechanical properties must be related to both high molecular weights and the structure. In addition, the [*η*] gradually decreased with the introduction of the PBTh segment, suggesting that it was more difficult to reach a high molecular weight in aromatic PBTh than in aliphatic PBS, probably due to the difference in the reactivity of PBTh-diol and PBS-diol.

### 3.2. Thermal Properties and Crystalline Morphology Studies

**Figure 5:**
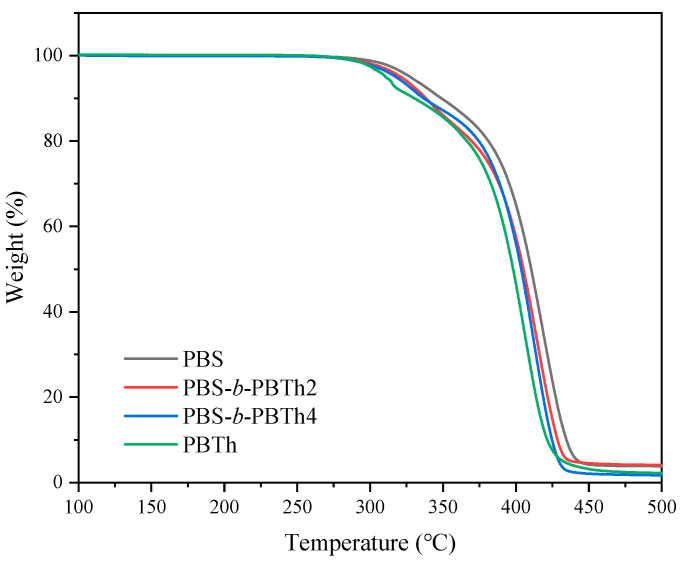
TGA curves of PBS, PBS-*b*-PBTh, and PBTh.

Most semicrystalline polymers are prepared into products through various melt processing techniques such as extrusion, molding, and blowing; therefore, the thermal stability must be carefully investigated from a polymer processing viewpoint. In this section, the thermal stability of PBS, PBS-*b*-PBTh, and PBTh was studied with TGA. As displayed in Figure 5, no obvious mass loss was detected before 250 °C, indicating that all samples showed relatively good thermal stability and could be treated through common polymer melt processing. The thermal decomposition temperature at 5% mass loss (*T*_d_) of PBS was about 328 °C, while that of PBTh was about 311 °C. The *T*_d_ values were about 322 and 318 °C for PBS-*b*-PBTh2 and PBS-*b*-PBTh4, respectively. Despite the differences in the chemical structure, composition, and intrinsic viscosity, the *T*_d_ was above 300 °C for all samples, showing a wide polymer processing window. It should also be noted that the *T*_d_ values of PBS and PBTh synthesized via a chain extension reaction were lower than those synthesized directly from two-stage melt polycondensation. For instance, the directly synthesized PBS showed a *T*_d_ of about 361 °C [10], while PBTh displayed a *T*_d_ of about 379 °C [26]. The decrease in *T*_d_ should be related to the decomposition of HDI at lower temperature [43]. Similar results were also reported in previous studies [38,39]. Figure S1 displays the corresponding derivative thermogravimetric (DTG) results, from which the temperature at the maximum mass loss rate (*T*_max_) values were read. The *T*_max_ of PBS was about 419 °C, while that of PBTh was only about 406 °C. For PBS-*b*-PBTh2 and PBS-*b*-PBTh4, the *T*_max_ values were 416 and 413 °C, respectively. Table S1 lists all the *T*_d_ and *T*_max_ values for comparison. It should be emphasized that these *T*_d_ and *T*_max_ values were heating rate dependent. In brief, the higher the heating rate, the greater the thermal values. As a result, the *T*_d_ and *T*_max_ values must be smaller if a slower heating rate of 10 °C/min is used.

**Figure 6:**
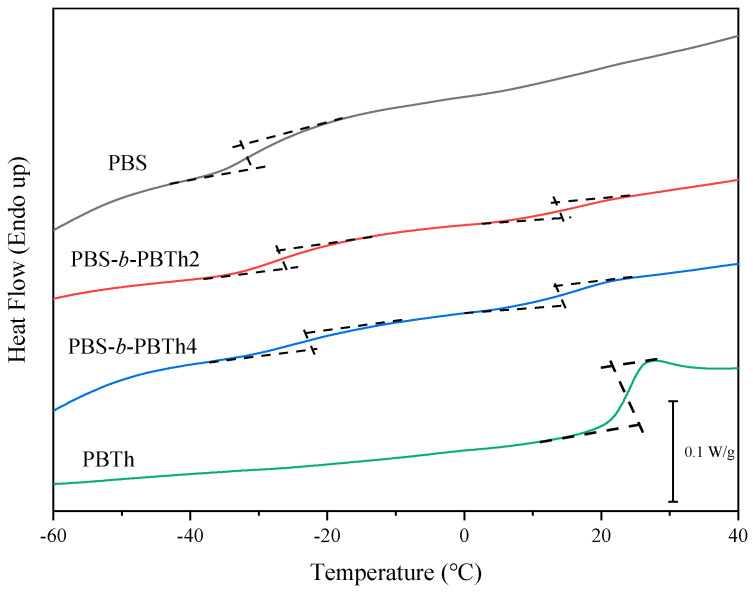
DSC heating curves at 10 °C/min showing the glass transition regions of PBS, PBS-*b*-PBTh, and PBTh after a quenching at 60 °C/min.

The basic thermal parameters were further investigated with DSC for PBS, PBS-*b*-PBTh, and PBTh. As described in the experimental section, the *T*_g_ values were first determined to study the phase behavior between the PBS and PBTh segments. Figure 6 illustrates the DSC heating curves, showing the glass transition regions of PBS, PBS-*b*-PBTh, and PBTh. As depicted in Figure 6, PBS showed a low *T*_g_ of −31.4 °C, while PBTh displayed a high one of 24.5 °C. In the case of PBS-*b*-PBTh, two separate *T*_g_ values were observed at low and high temperature ranges, corresponding to the *T*_g_ of the PBS segment and that of the PBTh segment, respectively. On the one hand, the *T*_g_ of the PBS soft segment gradually increased to −27.2 for PBS-*b*-PBTh2 and −21.9 °C for PBS-*b*-PBTh4 from −31.4 °C for PBS. On the other hand, the *T*_g_ of the PBTh hard segment gradually decreased to 17.9 for PBS-*b*-PBTh2 and 17.0 °C for PBS-*b*-PBTh4 from 24.5 °C for PBTh. As the two *T*_g_ values corresponding to the two segments approached each other in PBS-*b*-PBTh, this indicates that the two segments should be partially miscible in the amorphous region. In previous studies, the two segments were also partially miscible in PBS-*b*-PDEGT, while the two segments were miscible in the amorphous region in PBS-*b*-PBSe [38,39]. In brief, PBS-based multiblock copolyesters may show different phase behaviors depending on the choice of different hard or soft segments.

**Figure 7:**
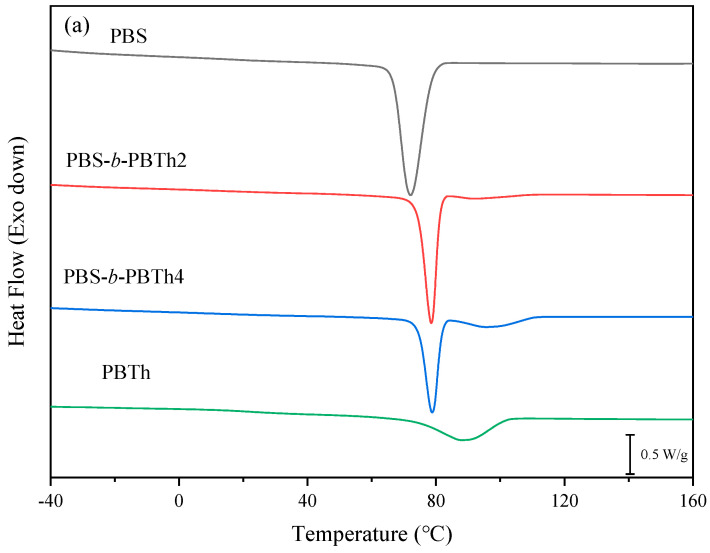

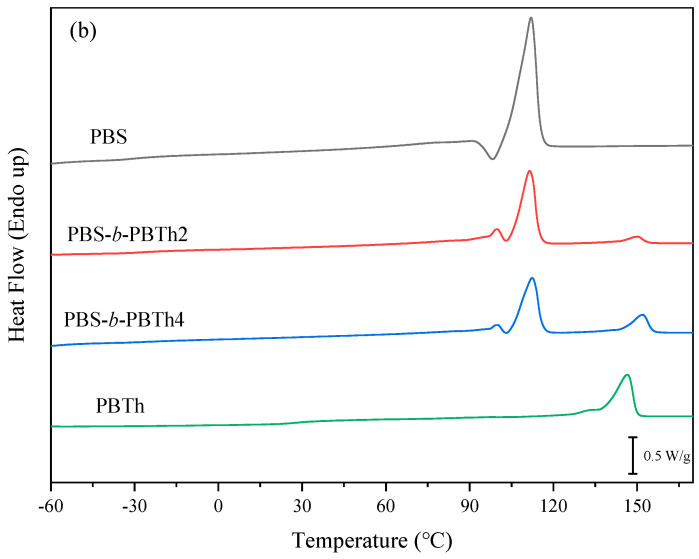
(**a**) DSC cooling curves at 10 °C/min and (**b**) subsequent heating curves at 10 °C/min of PBS, PBS-*b*-PBTh, and PBTh.

The effect of the PBTh segment on the melt crystallization and subsequent melting behavior of the PBS segment was further studied with DSC. Figure 7a displays the melt crystallization behavior, while Figure 7b illustrates the subsequent melting behavior. PBS showed a *T*_cc_ of 72.1 °C with a crystallization enthalpy (Δ*H*_cc_) of 76.1 J/g, while PBTh displayed a *T*_cc_ of 88.1 °C with a Δ*H*_cc_ of 31.1 J/g. The *T*_cc_ of PBS was lower than that of PBTh because the *T*_m_ of the former (114 °C) was obviously lower than that of the latter (150 °C) [3,25]. On the contrary, the Δ*H*_cc_ of PBS was remarkably higher than that of PBTh. In the case of PBS-*b*-PBTh, two distinct crystallization exothermic peaks were observed, corresponding to the separate crystallization of each segment. As seen in Figure 7a, a narrow and strong crystallization peak was found for the PBS segment at the low temperature range, while a broad and weak crystallization peak was discovered for the PBTh segment at the high temperature range. The *T*_cc_ of PBTh segment was higher in PBS-*b*-PBTh than in PBTh. For example, the *T*_cc_ of the PBTh segment increased slightly from 88.1 °C for PBTh to 92.7 °C for PBS-*b*-PBTh2 and 97.4 °C for PBS-*b*-PBTh4. The increased *T*_cc_ of the PBTh segment in PBS-*b*-PBTh may be related to the interface-assisted crystallization mechanism [44,45,46]. In the case of PBS-*b*-PBTh, both segments were partially miscible, displaying obvious phase separation and phase interface. The phase separation morphology is shown in the following crystalline morphology study section observed by POM. During the cooling process from the phase separation molten state, the crystallization of the high *T*_m_ component PBTh segment first occurred in the presence of the phase separation interface, which behaved as the substrates for the later heterogeneous nucleation of the PBTh segment [44,45,46]. Similar results were also reported in other blend or multiblock polymer systems [45,46]. The *T*_cc_ and Δ*H*_cc_ of the PBTh segment were both smaller in PBS-*b*-PBTh2 than in PBS-*b*-PBTh4 because of the reduced PBTh segment content.

In addition, the *T*_cc_ of the PBS segment was also higher in PBS-*b*-PBTh than in PBS. For instance, the *T*_cc_ of the PBS segment increased to 78.5 and 78.8 °C for PBS-*b*-PBTh2 and PBS-*b*-PBTh4, respectively, from 72.1 °C for PBS. It should be noted that the *T*_cc_ of the PBS segment for PBS-*b*-PBTh2 and PBS-*b*-PBTh4 was almost the same, although they were both about 6 °C higher than that of PBS. The increase in the *T*_cc_ of the PBS segment should arise from the preexisting PBTh crystals, which may provide more additional nucleation sites for the crystallization of the PBS segment. The POM study in the following section provides direct evidence for this assumption. It should also be emphasized that the enhanced crystallization of the PBS segment was seldom in PBS-based multiblock copolymers. In the case of PBS-*b*-PBSe and PBS-*b*-PDEGT systems, the presence of the other segment suppressed the crystallization of the PBS segment [38,39].

Figure 7b displays the subsequent melting behavior. PBS showed a *T*_m_ of 112.1 °C, while PBTh displayed a *T*_m_ of 146.6 °C. Two main melting peaks were observed for PBS-*b*-PBTh, with the lower one corresponding to the *T*_m_ of the PBS segment and the higher one corresponding to that of the PBTh segment. It should also be noted that double melting behavior occurred for the PBS segment in PBS-*b*-PBTh, which could be well-described by the melting, recrystallization, and remelting mechanism [47]. As seen in Figure 7b, the *T*_m_ values of the PBS segment was 111.5 and 112.4 °C for PBS-*b*-PBTh2 and PBS-*b*-PBTh4, respectively, which were almost the same as that of PBS (112.1 °C). The results indicated that the presence of the PBTh segment did not obviously affect the *T*_m_ of the PBS segment. However, the depression in *T*_m_ was very serious in the PBS random copolyesters, depending on the comonomer unit and ratio [13,14,15,16,17]. The *T*_m_ of the PBTh segment was even slightly higher in PBS-*b*-PBTh than in PBTh. The slight increase in *T*_m_ of the PBTh segment in PBS-*b*-PBTh may be related to the greater thickness of the lamellae formed at a relatively higher crystallization temperature. All of the related data from Figure 7a,b are summarized in Table 2.

**Figure 8:**
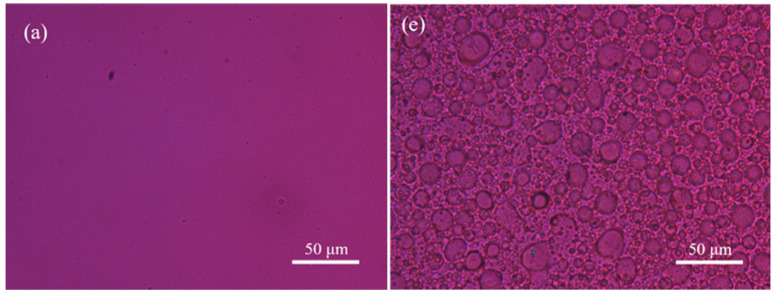

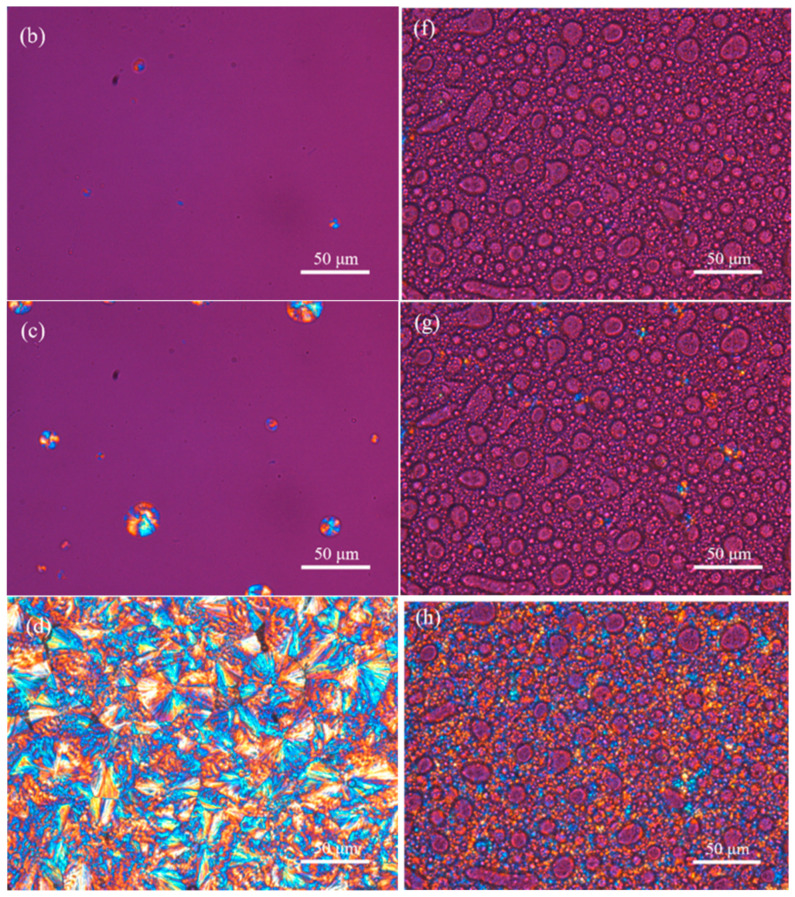
Crystalline morphology of (**a**) PBS at 115 °C, (**b**) PBS at 85 °C, (**c**) PBS at 80 °C, and (**d**) PBS at 70 °C; (**e**) PBS-*b*-PBTh4 at 140 °C, (**f**) PBS-*b*-PBTh4 at 90 °C, (**g**) PBS-*b*-PBTh4 at 85 °C, and (**h**) PBS-*b*-PBTh4 at 80 °C during a cooling process at a rate of 10 °C/min.

The crystalline morphology of PBS and PBS-*b*-PBTh4 was further investigated with POM to explore the effect of the PBTh segment on the crystallization of the PBS segment. Figure 8a–d illustrates the crystalline morphology of PBS, while Figure 8e,f displays the crystalline morphology of PBS-*b*-PBTh4. Both PBS and PBS-*b*-PBTh4 were crystallized during a cooling process at a rate of 10 °C/min from the completely molten state without any residual nuclei. As seen in Figure 8a, PBS remained in the molten state at 115 °C, which was close to the *T*_m_ of PBS. As the temperature decreased to 85 °C, which was lower than the *T*_m_ of PBS by almost 30 °C, Figure 8b shows that several spherulites appeared randomly. As seen in Figure 8c, more and more PBS spherulites were observed with a further decrease in temperature to 80 °C; moreover, the size of several PBS spherulites became larger due to the continuous growth during the crystallization process. Figure 8d shows that PBS spherulites filled the entire space at 70 °C.

The crystalline morphology evolution of the PBTh and PBS segments was also investigated with POM during a continuous cooling process at a rate of 10 °C/min from the molten state. As shown in Figure 8e, PBS-*b*-PBTh4 was also in the molten state at 140 °C, which was close to the *T*_m_ of PBTh. A distinct sea-island phase separation morphology was observed for PBS-*b*-PBTh4, with the PBS segment being the continuous phase while the PBTh segment was the dispersed phase. As displayed in Figure 8f, the crystallization of PBTh finished within the dispersed phase, while the crystallization of PBS started to occur in the continuous phase when the temperature decreased to 90 °C.

Figure 8g,h revealed that the crystallization of PBS continued to develop at 85 °C and was finally completed at 80 °C. The size of the PBS crystals was remarkably smaller in PBS-*b*-PBTh4, while the number of PBS crystals was significantly greater in PBS-*b*-PBTh4 than in PBS. It should also be noted that the PBS segment in PBS-*b*-PBTh4 finished crystallization at 80 °C, whereas PBS required a lower temperature and greater supercooling to achieve complete crystallization at 70 °C. During the cooling process, the high *T*_m_ component PBTh segment crystallized first; therefore, the preexisting PBTh crystals provided more additional nucleation sites for the crystallization of the low *T*_m_ component PBS segment. In conclusion, the above DSC and POM results indicate that the presence of the preexisting PBTh crystals favored the subsequent crystallization of the PBS segments.

### 3.3. Crystal Structure Study

**Figure 9:**
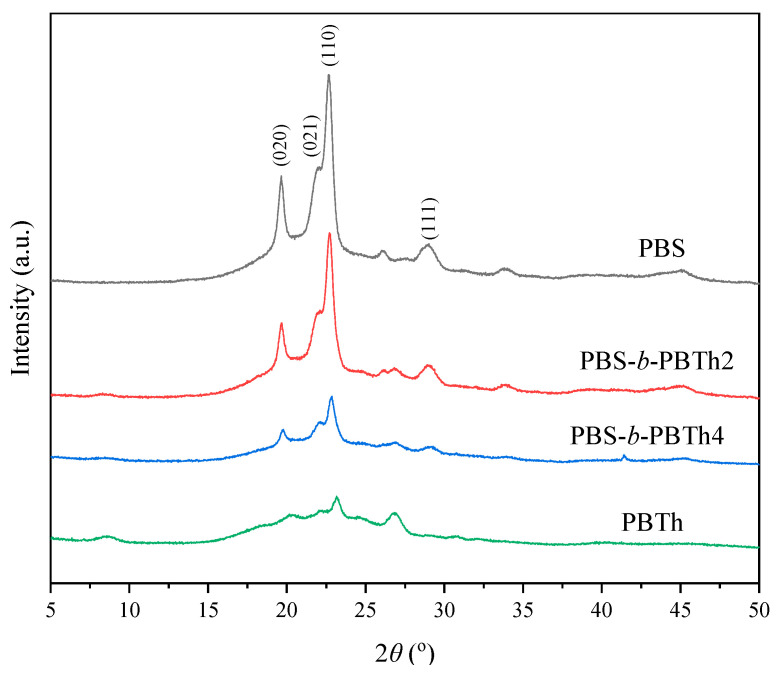
WAXD profiles of PBS, PBS-*b*-PBTh, and PBTh.

In the previous section, the thermal stability, crystallization and melting behaviors, and crystalline morphology of PBS-*b*-PBTh were systematically investigated with TGA, DSC, and POM to explore the effect of the incorporation of PBTh segment. In this section, the crystal structure of PBS-*b*-PBTh was further studied. Figure 9 shows the WAXD profiles of PBS, PBS-*b*-PBTh, and PBTh. For PBS, four major diffraction peaks were observed at 2θ = 19.6°, 21.9°, 22.7°, and 29.0°, corresponding to the (020), (021), (110), and (111) planes of PBS [48]. For PBTh, two relatively strong diffraction peaks appeared at 2θ = 23.1° and 26.9°, along with some relatively weak diffraction peaks at 2θ = 8.7°, 18.3°, 22.2°, and 24.5°. Lotti et al. reported that these peaks should correspond to the α-crystal form of PBTh, which is its most thermally stable polymorph [49]. For the PBS-*b*-PBTh block copolymers, the characteristic diffraction peaks of both the PBS and PBTh segments were observed simultaneously, demonstrating that the two components crystallized separately and formed distinct crystalline phases. This observation was consistent with the above crystallization and melting behavior results.

### 3.4. Mechanical Properties Study

**Figure 10:**
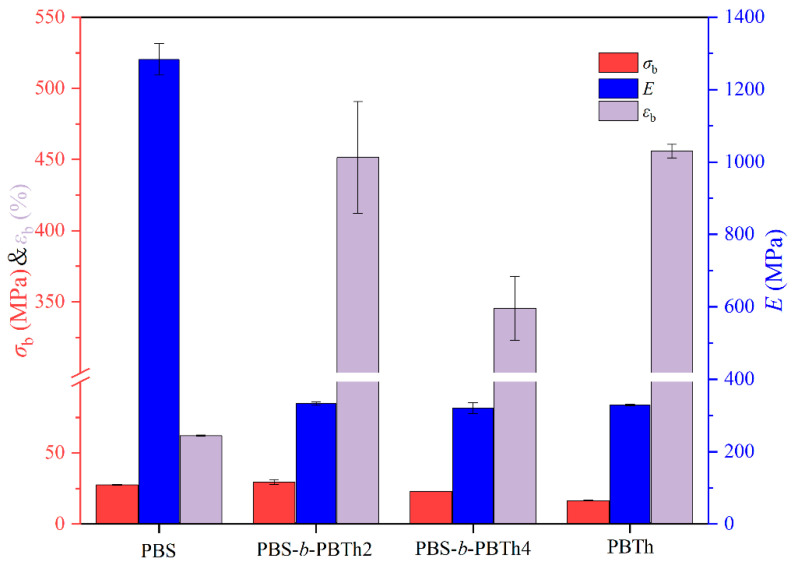
Tensile properties of PBS, PBS-*b*-PBTh, and PBTh.

The mechanical properties of PBS, PBTh, and PBS-*b*-PBTh were analyzed through tensile testing. Figure S2 depicts the stress–strain curves for all samples. Figure 10 illustrates the bar charts of the tensile strength (*σ*_b_), elongation at break (*ε*_b_), and Young’s modulus (*E*) for all samples. The detailed mechanical properties data are provided in Table S2. From Figure 10 and Table S2, PBS exhibited an *E* of 1283.5 ± 43.1 MPa, a *σ*_b_ of 27.6 ± 0.3 MPa, and a small *ε*_b_ of 62.1 ± 0.5%. On the contrary, PBTh showed a lower *E* of 328 ± 2.1 MPa, a smaller *σ*_b_ of 16.5 ± 0.3 MPa, and a higher *ε*_b_ of 455.9 ± 4.8%. The mechanical properties of PBTh were comparable to those of PBTh synthesized directly by Lotti et al., who reported an *E* of 89 ± 7 MPa, a *σ*_b_ of 24.5 ± 0.5 MPa, and a *ε*_b_ of 555 ± 50% [25]. The slight difference in the mechanical properties data of PBTh between the two labs could be related to the different synthesis method and molecular weights. Both PBTh samples displayed a relatively hard and tough mechanical behavior. After the addition of PBTh segments, the *E* value significantly decreased to 320.3 ± 15.1 MPa for PBS-*b*-PBTh4, comparable to that of PBTh. Moreover, the incorporation of the PBTh segments resulted in a substantial improvement in *ε*_b_. For instance, the *ε*_b_ of PBS-*b*-PBTh2 significantly increased to 451.5 ± 39.3%, which was also comparable to that of PBTh but remarkably higher than that of PBS. However, the *ε*_b_ of PBS-*b*-PBTh4 was slightly lower than that of PBS-*b*-PBTh2, probably due to the reduced intrinsic viscosity (1.38 dL/g for PBS-*b*-PBTh2 and 0.94 dL/g for PBS-*b*-PBTh4). It should be noted that the multiblock copolyesters containing the same PBTh components showed better mechanical properties than the random copolyesters PBSTh20 and PBSTh40 reported by Zhu et al. [34]. In conclusion, the addition of PBTh segments into PBS successfully enhanced the toughness without a significant reduction in strength, achieving the desired balance between toughness and strength.

## 4. Conclusions

In this research, we successfully synthesized novel biobased double crystalline PBS-*b*-PBTh multiblock copolyesters with excellent thermal and mechanical properties as well as their homopolyesters PBS and PBTh through a chain extension reaction using PBS-diol and PBTh-diol as the prepolymers and HDI as the chain extender. The features of PBS-*b*-PBTh can be summarized as follows. Both segments were semicrystalline, with the aliphatic PBS segment being the soft segment as a low *T*_m_ component while the aromatic PBTh segment was the hard segment as a high *T*_m_ component. The phase behavior studies by DSC and POM indicated that the PBS and PBTh segments were partially miscible in the amorphous region, as evidenced by two separate *T*_g_ values originating from each component; moreover, clear phase separation occurred in the molten state with the PBS phase being the continuous phase and the PBTh phase being the dispersed phase. During the cooling process from the melt, the high *T*_m_ component PBTh segment crystallized first, and the low *T*_m_ component PBS segment crystallized later. In addition, both the *T*_cc_ of the PBTh segment and that of the PBS segment were higher in PBS-*b*-PBTh than in PBTh and PBS due to different mechanisms. The increase in *T*_cc_ of the PBTh segment in PBS-*b*-PBTh was attributed to the interface-assisted crystallization mechanism, while the increase in *T*_cc_ of the PBS segment in PBS-*b*-PBTh was related to the nucleation agent effect of the preexisting PBTh crystals. During the subsequent melting behavior study, both the PBS and PBTh segment still maintained high *T*_m_ values, which were completely different from the obvious depression in *T*_m_ in the random copolymers. The WAXD study indicated that both segments crystallized separately and showed their own characteristic diffraction peaks. The tensile properties were further studied. PBS-*b*-PBTh displayed a significant increase in the elongation at break while still maintaining a relatively high break strength compared with those of PBS. In brief, novel biobased double crystalline PBS-*b*-PBTh multiblock copolymers were successfully synthesized in this research, which showed excellent thermal and mechanical properties and enhanced crystallization behavior. This study should be interesting and important in the field of biobased polymers.

## Figures and Tables

**Table 1:** Composition and intrinsic viscosity of PBS, PBS-*b*-PBTh multiblock copolyesters, and PBTh.

Samples	PBS		PBTh		HDI		[*η*] (dL/g)
	*f*_PBS_ (wt%)	*F*_PBS_ (wt%)	*f*_PBTh_ (wt%)	*F*_PBTh_ (wt%)	*f*_HDI_ (wt%)	*F*_HDI_ (wt%)	
**PBS**	96.6	97.3	/	/	3.4	2.7	1.44
PBS-*b*-PBTh2	77.4	78.9	19.4	18.6	3.2	2.5	1.38
PBS-*b*-PBTh4	58.2	55.9	38.8	40.1	3.0	4.0	0.94
PBTh	/	/	97.3	95.9	2.7	4.1	0.96

*f:* feed mass ratio; *F*: mass composition ratio calculated by ^1^H NMR spectra.

**Table 2:** Thermal properties of PBS, PBS-*b*-PBTh, and PBTh.

Samples	*T*_g1_ (°C)	*T*_g2_ (°C)	*T*_cc1_ (°C)	Δ*H*_cc1_ (J/g)	*T*_cc2_ (°C)	Δ*H*_cc2_ (J/g)	*T*_m1_ (°C)	*T*_m2_ (°C)
PBS	−31.4	/	72.1	76.1	/	/	112.1	/
PBS-*b*-PBTh2	−27.2	17.9	78.5	44.1	92.7	2.9	111.5	150.5
PBS-*b*-PBTh4	−21.9	17.1	78.8	33.8	97.4	8.7	112.4	152.1
PBTh	/	24.5	/	/	88.1	31.1	/	146.6

1: from PBS and 2: from PBTh.

## Data Availability

The original contributions presented in this study are included in the article/Supplementary Materials. Further inquiries can be directed to the corresponding author.

## Supplementary Materials

The following supporting information can be downloaded at: https://www.mdpi.com/article/10.3390/polym17040450/s1, Figure S1: DTG curves of PBS, PBS-b-PBTh, and PBTh; Figure S2: Stress-strain curve of PBS, PBTh, and PBS-b-PBTh; Table S1: Summary of the Td and Tmax values of PBS, PBS-b-PBTh, and PBTh; Table S2: Mechanical properties of PBS, PBS-b-PBTh, and PBTh.
