# Supplementary material for: Novel Biobased Double Crystalline Poly(butylene succinate)-b-poly(butylene 2,5-thiophenedicarboxylate) Multiblock Copolymers with Excellent Thermal and Mechanical Properties and Enhanced Crystallization Behavior
Source: Polymers (Basel). 2025 Feb 8;17(4):450. doi: 10.3390/polym17040450 (PMC11859257; doi:10.3390/polym17040450)
Supplement: Supplementary file 1 [file polymers-17-00450-s001.zip › polymers-3433372-supplementary.pdf]

Novel Biobased Double Crystalline Poly(butylene succinate)-*b*-poly(butylene 2,5-thiophenedicarboxylate) Multiblock Copolymers with Excellent Thermal and Mechanical Properties and Enhanced Crystallization Behavior

Haidong Yang, Shiwei Feng and Zhaobin Qiu *

State Key Laboratory of Chemical Resource Engineering, Beijing University of Chemical Technology,
Beijing 100029, China; 2023200346@grad.buct.edu.cn (H.Y.); 2023400126@grad.buct.edu.cn (S.F.)
* Correspondence: qiuzb@mail.buct.edu.cn

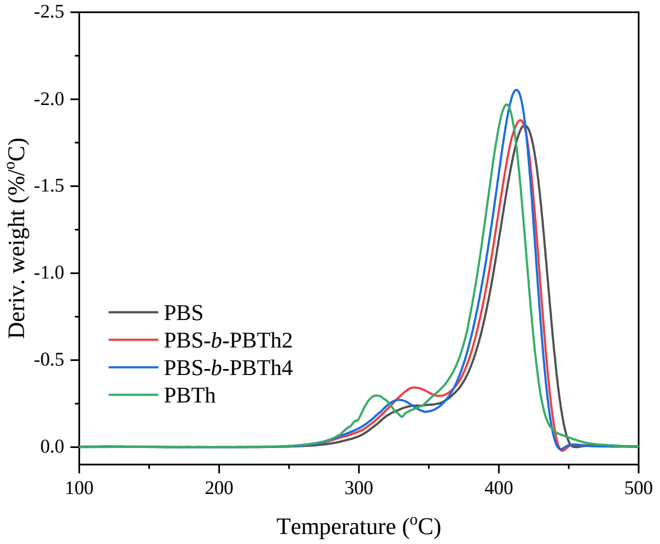

Figure S1. DTG curves of PBS, PBS-*b*-PBTh, and PBTh.

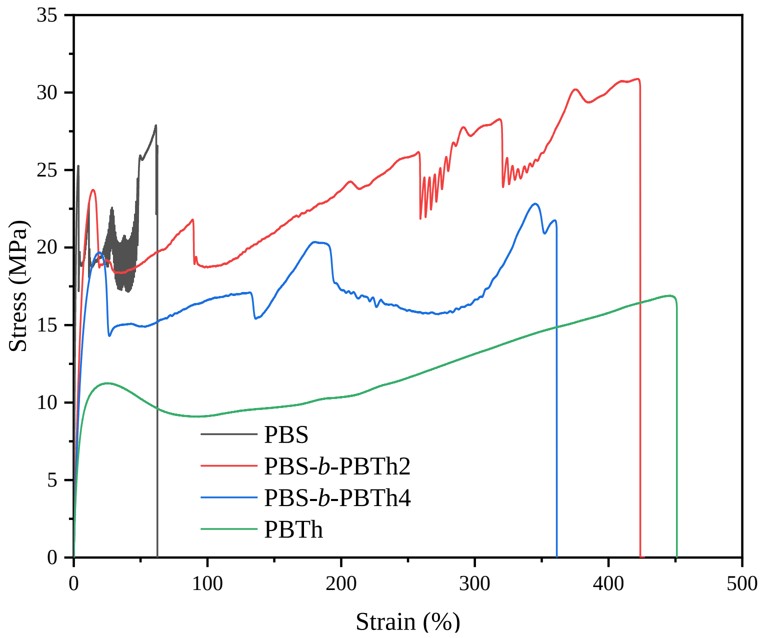

Figure S2. Stress-strain curve of PBS, PBTh, and PBS-*b*-PBTh.

Table S1. Summary of the T_d and T_{max} values of PBS, PBS-*b*-PBTh, and PBTh.

Samples	$T_{d5\%}$ (°C)	$T_{d50\%}$ (°C)	$T_{d92.5\%}$ (°C)	T_{max} (°C)
PBS	328	410	437	419
PBS-*b*-PBTh2	322	405	431	416
PBS-*b*-PBTh4	318	403	427	413
PBTh	311	398	425	406

$T_{d5\%}$, $T_{d50\%}$, and $T_{d92.5\%}$ were the thermal decomposition temperatures at 5%, 50%, and 92.5% mass loss, respectively.

Table S2. Mechanical properties of PBS, PBS-*b*-PBTh, and PBTh.

Samples	*E* (MPa)	σ_b (MPa)	ε_b (%)
PBS	1283.5 ± 43.1	27.6 ± 0.3	62.1 ± 0.5
PBS-*b*-PBTh2	333.1 ± 4.8	29.5 ± 1.8	451.5 ± 39.3
PBS-*b*-PBTh4	320.3 ± 15.1	22.9 ± 0.2	345.4 ± 22.3
PBTh	328 ± 2.1	16.5 ± 0.3	455.9 ± 4.8
